# Use of a sharps bin to provide lower limb traction

**DOI:** 10.1308/003588412X13373405385214b

**Published:** 2012-07

**Authors:** HA Kazi, TG Thomas

**Affiliations:** Wirral University Teaching Hospital NHS Foundation Trust,UK

**Figure 1:**
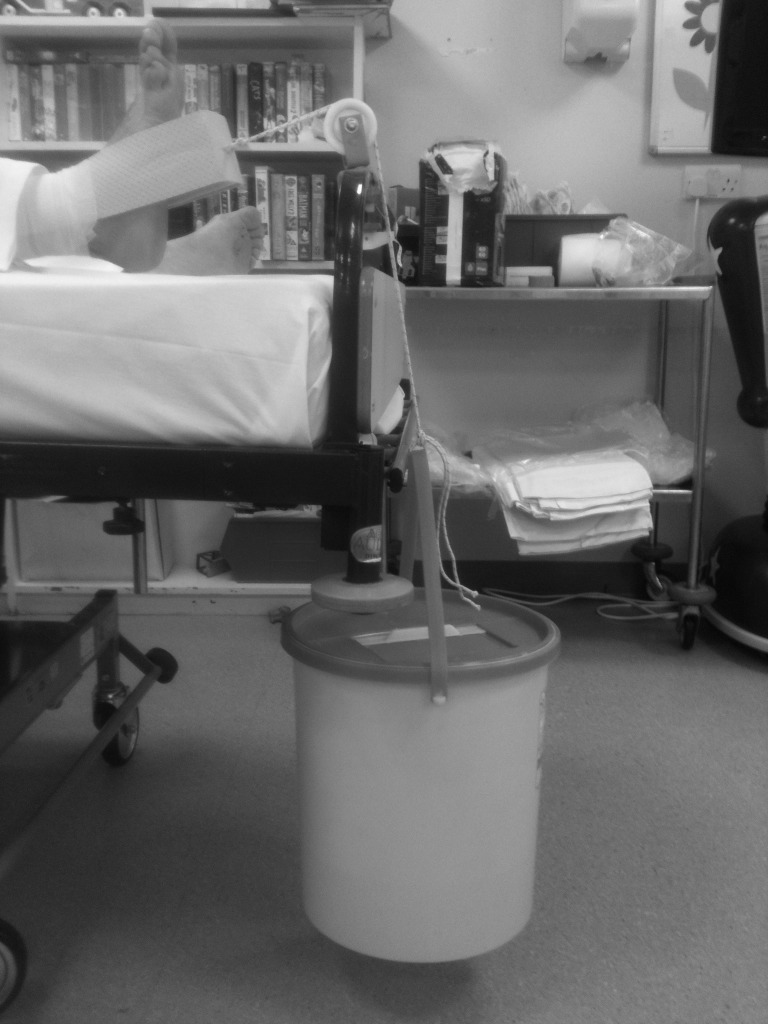
Sharps bin providing traction

Many lower extremity, pelvic and acetabular fractures require traction as first aid management prior to definitive fixation. While skin traction and Thomas splints are generally available, weights to provide countertraction are often missing or in parts of the hospital remote to the emergency department. A sharps bin (Sharpsguard® orange 11.5; Daniels, Oxford, UK) filled two-thirds with tap water and tied via its bucket handle to skin traction (Fig 1) provides approx 8kg of traction. This can effect reduction and temporary traction until weights are available.

